# Editorial: Portal Hypertension in Cirrhosis: From Pathogenesis to Novel Treatments

**DOI:** 10.3389/fphys.2022.864083

**Published:** 2022-03-21

**Authors:** Chandana B. Herath, Peter W. Angus, Jonel Trebicka

**Affiliations:** ^1^Ingham Institute for Applied Medical Research, South Western Sydney Clinical School, University of New South Wales, Liverpool, NSW, Australia; ^2^Department of Gastroenterology, Austin Health, University of Melbourne, Melbourne, VIC, Australia; ^3^Translational Hepatology, Medical Department, University of Frankfurt, Frankfurt, Germany

**Keywords:** cirrhosis, portal hypertension, pathophysiology of cirrhosis, novel treatment, portal vein thrombosis (PVT), transjugular intrahepatic portosystemic shunts, macrophage activation markers, liver stiffness measurement (LSM)

This Editorial summarizes the contributions to the Frontiers Research Topic “*Portal Hypertension in Cirrhosis: From Pathogenesis to Novel Treatments*” with peer-reviewed articles published in Frontiers in Physiology (Gastrointestinal Sciences) and Frontiers in Medicine (Gastroenterology).

Cirrhosis and its complications are responsible for a large number of deaths worldwide annually. Almost 90% of patients with cirrhosis eventually develop portal hypertension (PHT) and this condition is a prequel to the majority of deaths in these patients. Cirrhotic PHT results from increased intrahepatic vascular resistance combined with extrahepatic hyperdynamic circulatory state characterized by a high cardiac output and splanchnic vasodilatation. There have been very few new therapies introduced for the long-term management of PHT over the last 30 years. Thus, the goal of this Research Topic was to highlight recent advances in PHT research and in particular, novel concepts of pathophysiological pathways (e.g., transcription regulation, systemic inflammation), but also to remind the field of already known and possibly forgotten mechanisms (e.g., impact of abdominal surgery, role of bile acids) in the development and modulation of PHT. In addition, this Research Topic elaborates on new and established diagnostics and therapies in PHT.

Portal vein thrombosis (PVT) is an important complication of cirrhosis that aggravates PHT and variceal bleeding. In general, transjugular intrahepatic portosystemic shunts (TIPS) are used as a therapeutic option to establish a shunt between the hepatic vein and the portal vein to reduce portal pressure. Wang et al. conducted a retrospective analysis to study and compare the effectiveness of TIPS in cirrhotic patients with and without PVT and found that cirrhotic patients with PVT were equally able to tolerate TIPS. This suggests that this treatment may be used in the management of cirrhotic PHT with PVT. The Research Topic also included a paper by Chen et al. who conducted a retrospective study to investigate the impact of PVT on cirrhosis decompensation and survival of cirrhotic patients and found that PVT has no significant impact on the progression of cirrhosis.

Developing a novel drug to improve cirrhotic PHT by reducing hepatic vascular resistance to incoming portal blood flow whilst increasing splanchnic vascular resistance to lower mesenteric blood flow is a difficult task because vasoactive drugs can either vasodilate or vasoconstrict the respective vasculature. In this Research Topic, Zhao et al. comprehensively demonstrated that PTUPB, a dual cyclooxygenation-2 (COX-2) and soluble epoxy hydrolase (sEH) inhibitor, given orally to carbon tetrachloride-induced cirrhotic PHT rats markedly improved PHT by reducing hepatic extracellular matrix deposition and hepatic vascular remodeling while also increasing the wall thickness of the superior mesenteric artery which helped improve splanchnic vascular resistance. It was notable that PTUPB treatment caused a massive ~70% reduction in portal pressure from its baseline levels. The findings thus suggest that PTUPB which acts on both hepatic and splanchnic vasculatures is a potential drug candidate to treat cirrhotic PHT.

Macrophage activation plays a vital role in the pathogenesis of chronic liver disease and has also been linked to cirrhotic PHT, although a role in non-cirrhotic PHT has not been established. Ørntoft et al. evaluated the role of macrophage activation by measuring well-characterized markers of macrophage activation, soluble CD163 (sCD163) and soluble mannose receptor (sMR), in cohorts of patients with idiopathic PHT, non-cirrhotic patients with PVT and compared with those of cirrhotic patients with and without PVT and healthy subjects. The findings that elevated levels of sCD163 and sMR in cirrhotic patients with or without PVT compared to low levels of the markers in patients with idiopathic PHT and in non-cirrhotic PVT patients suggested that hepatic macrophage activation with elevated sCD163 and sMR levels is closely linked to the underlying liver disease with cirrhosis rather than PHT.

Hepatic surgery is generally contraindicated in patients with advanced liver disease since it increases the chances of acute decompensation of cirrhosis and multiorgan failure, resulting in high mortality. Similarly, extrahepatic surgical procedures have also been recognized as a main cause of mortality in cirrhotic patients with PHT. To further understand this, Chang et al. investigated the pathophysiology of post-operative decompensation of cirrhosis in cirrhotic animals undergoing extrahepatic intestinal manipulation (IM). The authors reported that IM significantly elevated portal pressure, induced systemic inflammation, and accelerated progression of liver fibrosis in the presence of liver injury. The findings thus suggest that these models may be useful to investigate pathophysiology of post-operative decompensation of cirrhosis which may be prevented by controlling portal pressure peri-operatively.

The formation of esophageal varices and variceal bleeding constitutes a major clinical manifestation of PHT in decompensated cirrhosis and has high associated morbidity and mortality. Although liver stiffness measurement (LSM) is an accurate widely used non-invasive tool for the diagnosis of liver fibrosis, its use to predict the occurrence of complications of liver cirrhosis such as esophageal variceal rebleeding in cirrhotic PHT patients has not been reported. In this Research Topic, Liu et al. conducted a prospective study to examine the effectiveness of LSM in predicting rebleeding compared with other non-invasive methods in hepatitis B cirrhotic patients. The authors reported that in comparison with other non-invasive methods including AST to Platelet Ratio Index, Fibrosis-4 score, King's College Criteria, Goteborg University Cirrhosis Index, Fibroindex, Fornsindex and transient elastography, LSM showed a highly reliable prediction performance of variceal rebleeding. The findings thus suggest that LSM can simply and accurately predict variceal rebleeding events in hepatitis B cirrhotic patients with PHT.

Balvey and Fernandez provided a comprehensive review of the literature on “translational control of liver disease,” highlighting the abnormalities in the regulation of translation of key mRNA transcripts by RNA-binding proteins during chronic liver disease and their pathological impact on PHT, fibrosis, steatosis, neovascularization, and cancer development. In a second review, Sauerbruch et al. analyzed the current literature to evaluate a possible vasoactive role of bile acids in the cirrhotic hyperdynamic circulatory state, based primarily on *in-vitro* studies, and suggested that long-term RCTs with hemodynamic endpoints are needed in patients with early-stage cirrhosis.

Finally, a case-report of subtotal splenectomy during auxiliary partial orthotopic liver transplantation has been published by Zhou et al. which demonstrated that this is a viable procedure for modulating portal inflow and correcting severe hypersplenism in patients with end-stage liver cirrhosis.

Collectively, whilst the original work published in this Frontiers Research Topic highlighted novel aspects of pathophysiology, diagnosis and treatment of cirrhotic PHT, potential limitations include the lack of mechanistic aspects of the work, for example, the mechanism(s) by which PTUPB improves PHT and investigation of regional blood flows using approaches such as fluorescent-labeled colored microsphere beads in animal models with cirrhotic PHT. Work on these areas potentially leads to future Research Topics.

## Author Contributions

All authors listed have made a substantial, direct, and intellectual contribution to the work and approved it for publication.

## Funding

This study was supported by grant funding to CH and PA from the National Health and Medical Research Council (NHMRC) of Australia (APP1062372 and APP1124125), the German Research Foundation (DFG) project ID 403224013 - SFB 1382 (A09), the German Federal Ministry of Education and Research (BMBF) for the DEEP-HCC project and by the Hessian Ministry of Higher Education, Research and the Arts (HMWK) for the ENABLE and ACLF-I cluster projects. The MICROB-PREDICT (project ID 825694), DECISION (project ID 847949), GALAXY (project ID 668031), LIVERHOPE (project ID 731875), and IHMCSA (project ID 964590) projects have received funding from the European Union's Horizon 2020 research and innovation program. The manuscript reflects only the authors' views, and the European Commission is not responsible for any use that may be made of the information it contains. The funders had no influence on study design, data collection and analysis, decision to publish, or preparation of the manuscript.

## Conflict of Interest

JT has received speaking and/or consulting fees from Versantis, Gore, Bayer, Alexion, Norgine, Grifols, and CSL Behring. The remaining authors declare that the research was conducted in the absence of any commercial or financial relationships that could be construed as a potential conflict of interest.

## Publisher's Note

All claims expressed in this article are solely those of the authors and do not necessarily represent those of their affiliated organizations, or those of the publisher, the editors and the reviewers. Any product that may be evaluated in this article, or claim that may be made by its manufacturer, is not guaranteed or endorsed by the publisher.

